# Cardioprotective Effect of Metformin Treatment in Mouse Model of Experimental Autoimmune Myocarditis

**DOI:** 10.1155/cdr/6615227

**Published:** 2026-10-12

**Authors:** Magdalena Czepiec, Katarzyna Fryt, Agnieszka Jaźwa-Kusior, Andrea Laimbacher, Gabriela Kania, Maciej Siedlar, Małgorzata Tyszka-Czochara, Przemysław Błyszczuk

**Affiliations:** ^1^ Department of Clinical Immunology Faculty of Medicine Jagiellonian University Medical College Cracow Poland cm-uj.krakow.pl; ^2^ Department of Medical Biotechnology Faculty of Biochemistry Biophysics and Biotechnology Jagiellonian University Cracow Poland uj.edu.pl; ^3^ Department of Rheumatology University Hospital Zurich University of Zurich Zurich Switzerland uzh.ch; ^4^ Department of Food Chemistry and Nutrition Faculty of Pharmacy Jagiellonian University Medical College Kraków Poland cm-uj.krakow.pl

**Keywords:** cardioprotection, dilated cardiomyopathy, experimental autoimmune myocarditis mouse model, metformin, myocarditis

## Abstract

**Background:**

Metformin is a well‐established treatment for Type 2 diabetes. Beyond its role in glycemic control, metformin has gained attention for its pleiotropic effects, including cardioprotective properties. However, its role in autoimmune myocarditis and subsequent postinflammatory dilated cardiomyopathy remains poorly understood.

**Methods:**

To investigate the role of metformin in autoimmune‐mediated cardiac disease, we employed the experimental autoimmune myocarditis mouse model. Six to eight weeks old Balb/c mice were immunized twice with an *α*‐myosin heavy chain peptide emulsified in complete Freund′s adjuvant to induce myocarditis and progression toward dilated cardiomyopathy. Metformin was administered in two approaches: a preventive (from Days 0 to 21 or 40) and a therapeutic (from Days 21 to 40). Cardiac function and disease progression were evaluated at two time points: Day 21 (inflammatory phase) and Day 40 (fibrotic phase), using echocardiography, histopathological assessment, and molecular analyses.

**Results:**

Treatment with metformin resulted in activation of the AMP‐activated protein kinase‐dependent signaling pathway during the inflammatory phase of myocarditis. This treatment was associated with activation of apoptosis‐related pathways and a moderate reduction in inflammatory cell infiltration. Mice receiving metformin in the preventive approach exhibited preserved cardiac function. This was accompanied by decreased cardiomyocyte cross‐sectional area and altered expression of hypertrophy‐associated genes (*Acta1* and *Myh6*). In contrast, neither preventive nor therapeutic administration of metformin significantly reduced the extent of myocardial fibrosis.

**Conclusion:**

Our findings suggest that metformin provides cardioprotective effects during the early inflammatory stages of autoimmune myocarditis and, to a lesser extent, during the later fibrotic remodeling phase.

**Significance Statement:**

Untreated acute myocarditis can lead to functional decline and postinflammatory dilated cardiomyopathy. Using an autoimmune myocarditis mouse model, we demonstrate that preventive metformin treatment reduces cardiac inflammation and preserves cardiac function. These findings suggest that metformin, in addition to its applications in diabetes, could be used to prevent inflammatory heart disease.

## 1. Introduction

Metformin is a well‐established and the most commonly used oral hypoglycemic drug for the treatment of Type 2 diabetes mellitus. As a member of the biguanide group, its mechanism of action primarily involves the reduction of hepatic glucose production and the improvement of tissue insulin sensitivity [1]. Among the proposed molecular mechanisms, activation of the AMP‐activated protein kinase (AMPK) signaling pathway has emerged as a central regulator of energy metabolism and cellular stress responses [2]. Beyond its role in glycemic control, metformin is increasingly recognized for its pleiotropic effects in various other pathological conditions. These include neurodegenerative diseases [3, 4], inflammatory disorders [5, 6], polycystic ovary syndrome [7, 8], and even cancer [9, 10]. Additionally, metformin has been associated with antiaging properties [11, 12] and, notably, cardioprotective effects. In light of these findings, numerous clinical trials have been initiated to evaluate the cardioprotective potential of metformin in both diabetic patients with coexisting cardiovascular disease (e.g., NCT00513630) and nondiabetic individuals at risk of cardiovascular events (e.g., NCT01217307, NCT05177588). Collectively, the outcomes of these trials indicate that metformin treatment reduces the occurrence of major cardiovascular events [13], with potential mechanisms including lowering of LDL cholesterol and large LDL particle levels [14], decreasing myocardial oxygen consumption [15], and reducing oxidative stress [16]. Preclinical studies in experimental animal models have further confirmed that metformin protects the heart against ischemic injury [17, 18], cardiac hypertrophy [19], and obesity‐related cardiac injury [20]. Moreover, a recent experimental study demonstrated that metformin attenuated endotoxin‐induced acute myocarditis through AMPK activation, highlighting its potential relevance in inflammatory heart damage [21]. However, to date, no studies have demonstrated a cardioprotective role of metformin in autoimmune myocarditis or in the development of postinflammatory dilated cardiomyopathy (DCM).

Inflammatory heart disease, also known as myocarditis, is induced by either infectious agents (e.g., viruses, bacteria, and protozoa) or noninfectious causes, such as autoimmune disorders or toxic medicaments [22, 23]. It is characterized by extensive infiltration of inflammatory cells into the myocardium and presents with nonspecific symptoms such as dyspnea, chest pain, and impaired left ventricular function. If left untreated, myocarditis may progress to DCM due to maladaptive remodeling, including myocardial fibrosis [24]. Fibrosis involves excessive accumulation of extracellular matrix components, particularly collagen Types I and III. This process results from an imbalance between matrix metalloproteinases and their tissue inhibitors, leading to scar formation. Consequently, myocardial wall thickening and stiffness impair both systolic and diastolic function, as well as electrical conduction [25–27]. Although the overall mortality rate of myocarditis is relatively low, severe forms may lead to life‐threatening complications, including DCM. Clinical data indicate that in patients with DCM, the 5‐year survival rate ranges from approximately 60%–80%, whereas the 10‐year survival rate can decline to even 30% [28].

Many animal models are currently available for researchers to review various heart disorders. One of them is the experimental autoimmune myocarditis (EAM) model in mice, which effectively mimics autoimmune‐mediated myocarditis caused by noninfectious triggers [29]. In this model, CD4^+^ T cells have been identified as primary initiators of cardiac‐specific autoimmunity and are often associated with heart‐reactive autoantibodies, particularly against *α*‐myosin heavy chain (*α*MyHC) [30–34]. This autoimmune response arises due to defective central tolerance mechanisms, specifically the failure of negative selection of *α*MyHC‐reactive CD4^+^ T cells in the thymus. In both mice and humans, absence of *α*MyHC expression in medullary thymic epithelial cells leads to a breakdown of self‐tolerance and promotes the development of heart‐specific autoimmunity [35–38]. In the EAM model, *α*MyHC peptide emulsified in complete Freund’s adjuvant (CFA) is repeatedly injected into Balb/c mice to trigger myocarditis. In consequence, the phase of acute myocarditis occurs, which is characterized by the infiltration of inflammatory cells. In the next few days, inflammation is replaced by fibrotic tissue deposition, resulting in myocardial hypertrophy, ventricular remodeling, and deterioration of cardiac function, leading to DCM phenotype [39]. Importantly, susceptibility to autoimmune myocarditis is strongly affected by the genetic background of the mice [40, 41]. Nevertheless, the EAM model continues to represent a reliable and reproducible experimental system for studying the immunopathological mechanisms of myocarditis and its progression toward DCM.

In this study, we employed the EAM model to evaluate the effects of metformin administered both preventively and therapeutically, aiming to investigate its potential cardioprotective properties in myocarditis and subsequent DCM, independent of its glucose‐lowering action.

## 2. Methods

### 2.1. EAM Mouse Model

All experimental procedures were conducted in accordance with national regulations on animal welfare and approved by the Local Ethics Committee for Animal Experiments (Permit No. 586/2021). The study adhered to the guidelines outlined in Directive 2010/63/EU of the European Parliament and of the Council on the protection of animals used for scientific purposes.

To induce EAM, Balb/c mice (male and female, 6–8 weeks old) were immunized subcutaneously on Days 0 and 7 with a heart‐specific synthetic *α*‐MyHC614‐634 peptide (Ac‐RSLKLMATLFSTYASADR, Caslo, Denmark) at dose: 200 *μ*g/injection/mice as described previously [40–43]. The peptide was emulsified in CFA (Sigma‐Aldrich, United States) in a 1:1 ratio, and injections were performed subcutaneously in the bilateral inguinal region (left and right sides) with a total injection volume of approximately 200 *μ*L per mouse for each immunization. Following random allocation into experimental groups, mice received metformin (Thermo Fisher Scientific, United States) administered ad libitum in drinking water at a concentration of 1.25 mg/mL. The estimated metformin dose was approximately 250 mg/kg/day, calculated based on average daily water consumption and body weight measurements. The dosing regimen was selected based on previous studies [44–46]. Control animals received regular drinking water without metformin. To assess the effect of metformin in the EAM model, two treatment regimens were employed: preventive and therapeutic. In the preventive protocol, metformin administration commenced on Day 0 and continued through Day 21 or Day 40. In the therapeutic protocol, treatment began on Day 21, after the onset of inflammation, and continued until Day 40. For the experiment with analysis at Day 21, both male and female mice (vehicle group *n* = 10, metformin group *n* = 9) were included. For the experiment with analysis at Day 40, only female mice were included (vehicle group *n* = 8, metformin‐treated in preventive approach *n* = 7, metformin‐treated in therapeutic approach *n* = 8). Animals were maintained under controlled environmental conditions with ad libitum access to food and water. Mice were euthanized by cervical dislocation at two designated time points: Day 21 (acute myocarditis phase) and Day 40 (postinflammatory DCM phase). Hearts were excised, washed in phosphate‐buffered saline (PBS, Gibco, United States), and stored at –80°C or in formalin for subsequent histological, molecular, or biochemical analyses.

### 2.2. Histological and Immunohistochemical Analysis

Mouse heart tissues were harvested at experimental endpoints (Days 21 and 40), fixed in 4% neutral‐buffered formalin, and subsequently embedded in paraffin. Tissue sections were cut using a microtome and mounted on glass slides for histological and immunohistochemical analyses. To assess cardiac inflammation at Day 21 of EAM, sections were stained with hematoxylin and eosin (H&E, Sigma‐Aldrich, United States). Myocarditis severity was scored on H&E sections using a semiquantitative 0–5 scale as described previously [42]. Cardiac fibrosis was assessed at Day 40 using Masson′s trichrome (Sigma‐Aldrich, United States) and Sirius Red (Abcam, United Kingdom) staining. Fibrotic areas were quantified and expressed as a percentage of the total myocardial area per tissue section. To evaluate cardiomyocyte hypertrophy, wheat germ agglutinin (WGA) staining conjugated with Alexa Fluor‐488 (Invitrogen, United States) and DAPI (Thermo Fisher Scientific, United States) was performed. Cardiomyocyte cross‐sectional area was measured in square micrometers (*μ*m^2^). Immunohistochemical staining was carried out to detect CD3^+^, CD45^+^ cells, and vimentin‐expressing cells. The following primary antibodies were used: anti‐CD3 (clone SP7, Neomarkers, United States), anti‐CD45 (clone A20, BD Biosciences, United States), and antivimentin (clone V9, Sigma‐Aldrich, United States). Detection was performed using HRP‐conjugated secondary antibodies and developed with diaminobenzidine (DAB) chromogen. Positive cells (CD3^+^, CD45^+^) were quantified as the number of immunopositive cells per mm^2^, whereas vimentin expression was expressed as the percentage of positive area within the total heart section. All stained sections were analyzed using ImageJ software (Version 1.52a, NIH, United States) and ImageScope software (Version 12.3.3, Leica Biosystems Imaging, Germany).

### 2.3. RNA Isolation and Quantitative Real‐Time PCR (qPCR)

Total RNA was isolated from mouse heart tissue using QIAzol Lysis Reagent (Qiagen, Germany), following the manufacturer′s protocol. Briefly, samples were lysed directly in QIAzol and homogenized using a TissueLyser II system (Qiagen, Germany). After homogenization and phase separation, RNA was precipitated and purified according to standard procedures. Complementary DNA (cDNA) was synthesized using the NG dART RT kit (EURx, Poland). qPCR was performed using SYBR Green PCR Master Mix (EURx, Poland) on a QuantStudio 7 Real‐Time PCR System (Applied Biosystems, United States). Transcript levels of elongation factor 2 (*Ef2*) were used as an endogenous housekeeping control. Relative gene expression levels were calculated using the 2^−*ΔΔ*Ct^ method. The following oligonucleotide primers were used in this study: *Acta1* 5 ^′^‐CAGAGCAAGCGAGGTATCC‐3 ^′^ and 5 ^′^‐GTCCCCAGAATCCAACACG‐3 ^′^, *Acta2* 5 ^′^‐CTCTCTTCCAGCCATCTTTCAT‐3 ^′^ and 5 ^′^‐TATAGGTGGTTTCGTGGATGC‐3 ^′^, *Bax* 5 ^′^‐CTCAAGGCCCTGTGCACTAAAGTG‐3 ^′^ and 5 ^′^‐CAGCCACCCTGGTCTTGGATC‐3 ^′^, *Bcl2* 5 ^′^‐GGGATGCCTTTGTGGAACTATATGGC‐3 ^′^ and 5 ^′^‐GGCAGGTTTGTCGACCTCACTTG‐3 ^′^, *Col1a1* 5 ^′^‐CCGCTGGTCAAGATGCTC‐3 ^′^ and 5 ^′^‐CTCCAGCCTTTCCAGGTTCT‐3 ^′^, *Col3a1* 5 ^′^‐TGACTGTCCCACGTAAGCAC‐3 ^′^ and 5 ^′^‐GAGGGCCATAGCTGAACTGA‐3 ^′^, *Ef2* 5 ^′^‐TTCATGATGTGACCCTGCAT‐3 ^′^ and 5 ^′^‐GGTCAGCACACTGGCATACA‐3 ^′^, *Il1b* 5 ^′^‐ATGAAGGGCTGCTTCCAAAC‐3 ^′^ and 5 ^′^‐ATGTGCTGCTGCGAGATTTG‐3 ^′^, *Il6* 5 ^′^‐TAGTCCTTCCTACCCCAATTTCC‐3 ^′^ and 5 ^′^‐TTGGTCCTTAGCCACTCCTTC‐3 ^′^, *Myh6* 5 ^′^‐GCCCAGTACCTCCGAAAGTC‐3 ^′^ and 5 ^′^‐ATCAGGCACGAAGCACTCC‐3 ^′^, *Myh7b* 5 ^′^‐CATGGGATGGTAAGAAACGGG‐3 ^′^ and 5 ^′^‐TCCTCCAGTAAGTCGAAACGG‐3 ^′^, *Nppb* 5 ^′^‐TGGGGAGGCGAGACAAG‐3 ^′^ and 5 ^′^‐AGCCCAAACGACTGACG‐3 ^′^, *Postn* 5 ^′^‐AGACTGCTTCAGGGAGACAC‐3 ^′^ and 5 ^′^‐ACGGCCTTCTCTTGATCGTC‐3 ^′^, *Ppargc1a* 5 ^′^‐CATTTGATGCACTGACAGATGGA‐3 ^′^ and 5 ^′^‐CCGTCAGGCATGGAGGAA‐3 ^′^, *Prkka1* 5 ^′^‐GACCGGACATAAAGTGGCTGT‐3 ^′^ and 5 ^′^‐CCACGTCAAGGCTCCGAAT‐3 ^′^, *Prkaa2* 5 ^′^‐ATGGTTGTCCATAGGGACCTG‐3 ^′^ and 5 ^′^‐GCGATCCACAGCTAGTTCGTAG‐3 ^′^, *Tnf* 5 ^′^‐TAGCCCACGTCGTAGCAAAC‐3 ^′^ and 5 ^′^‐ACAAGGTACAACCCATCGGC‐3 ^′^.

### 2.4. Echocardiography

Mice were anesthetized in an induction chamber containing 5% isoflurane (Aerrane, Baxter) delivered in air at a flow rate of 2 L/min. Following induction, they were positioned on a warming platform to maintain a body temperature of 37°C. A scavenger mask that supplied 1.5%–2% isoflurane in air at a flow rate of 2 L/min was used to maintain general anesthesia. Transthoracic echocardiographic assessment was performed using a Vevo 2100 Imaging System (VisualSonics, Canada) equipped with a 30‐MHz transducer. Two‐dimensional brightness mode (B‐mode) imaging was used to visualize the heart in a parasternal long‐axis (PLAX) a short‐axis (SAX), and an apical four‐chamber (A4C) views. The M‐mode imaging was applied in SAX view to evaluate left ventricular dimensions during systole and diastole periods. Diastolic function of the heart was obtained from A4C view using pulsed‐wave Doppler. The following functional and structural cardiac parameters were acquired and analyzed: heart rate (HR; beats per minute [BPM]), ejection fraction (EF; %), fractional shortening (FS; %), stroke volume (SV; *μ*L), cardiac output (CO; mL/min), left ventricular end‐diastolic diameter (LVID d; mm), left ventricular end‐systolic diameter (LVID s; mm), left ventricular end‐diastolic volume (LV Vol d; *μ*L), left ventricular end‐systolic volume (LV Vol s; *μ*L), left ventricular end‐diastolic posterior wall thickness (LVPW d; mm), left ventricular end‐systolic posterior wall thickness (LVPW s; mm), left ventricular end‐diastolic anterior wall thickness (LVAW d; mm), left ventricular end‐systolic anterior wall thickness (LVAW s; mm), peak Doppler blood inflow velocity across the mitral valve during early diastole (E; mm/s), peak Doppler blood inflow velocity across the mitral valve during late diastole (A; mm/s).

### 2.5. Statistical Analysis

Experimental data are presented as mean ± standard error of the mean (SEM). The normality of the data distribution was evaluated using the Shapiro–Wilk test, and the homogeneity of variances was assessed using the Brown–Forsythe test. For real‐time qPCR analyses, the ROUT method was additionally employed to identify and exclude statistical outliers. For normally distributed (parametric) data, unpaired Student′s *t*‐test or one‐way analysis of variance (ANOVA) was used. For nonnormally distributed (nonparametric) data, the Mann–Whitney *U* test or Kruskal–Wallis test was applied. Dunnett′s multiple comparisons test (for parametric ANOVA) and Dunn′s multiple comparisons test (for nonparametric Kruskal–Wallis test) were used for post hoc analysis. A *p* value < 0.05 was considered statistically significant. All statistical analyses were performed using GraphPad Prism 6 software (GraphPad Software, United States).

## 3. Results

### 3.1. Metformin Moderately Attenuates Myocarditis and Immune Infiltration

To assess the immunomodulatory and potential cardioprotective effects of metformin during the acute inflammatory phase of autoimmune myocarditis, we employed the EAM model in wild‐type Balb/c mice. EAM was induced by subcutaneous immunization with *α*MyHC/CFA on Days 0 and 7. Mice received either metformin dissolved in drinking water or regular drinking water from Days 0 to 21, which represents the acute phase of myocardial inflammation (Figure 1A). At Day 21 of EAM, no significant differences were detected between the metformin‐treated and control groups in terms of heart weight to body weight ratio (HW/BW) or heart weight to tibia length ratio (HW/TL), suggesting that metformin administration did not induce gross alterations in cardiac mass (Figure 1B). H&E‐based histopathological evaluation suggested a trend toward attenuated myocarditis severity in mice receiving metformin. Immunohistochemical staining for CD3^+^ T lymphocytes and CD45^+^ leukocytes confirmed a modest, though not statistically significant, reduction in immune cell presence compared with controls (Figure 1C).

**Figure 1:**
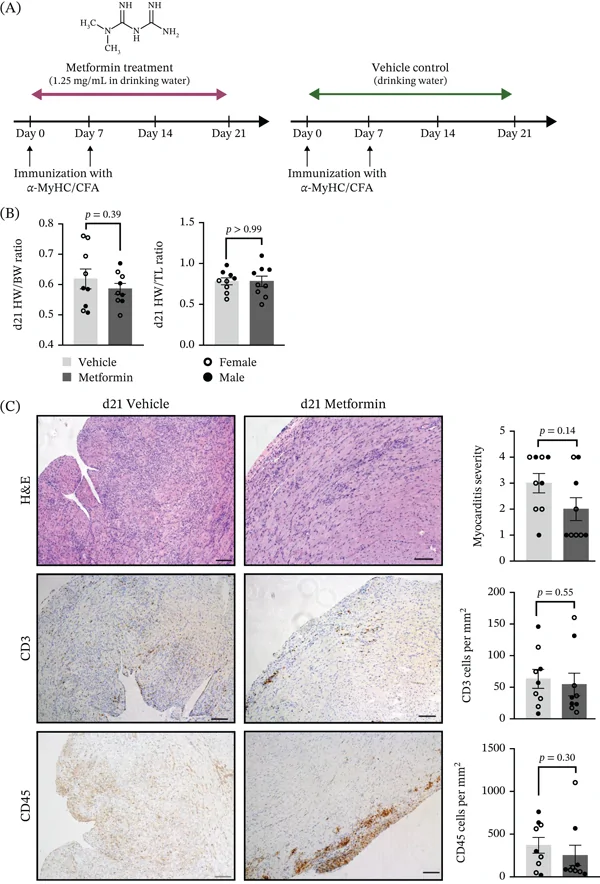
Preventive metformin modestly attenuates cardiac inflammation in autoimmune myocarditis. (A) Schematic representation of the experimental design. (B) Heart weight (HW) to body weight (BW) ratio and heart weight (HW) to tibia length (TL) ratio. (C) Representative histology of hematoxylin and eosin (H&E) staining and immunohistochemical staining for CD3 and CD45 along with quantification of myocarditis severity and number of CD3^+^ and CD45^+^ cells per mm^2^. Scale bars = 100 *μ*m. The values are expressed as mean ± SEM, with each dot representing an individual mouse (*n* = 9–10). *p* values were calculated using unpaired Student′s *t*‐test, except for myocarditis severity, CD3^+^ and CD45^+^ cells per mm^2^, which were analyzed with the nonparametric Mann–Whitney test.

To substantiate these findings at the molecular level, we performed qPCR analysis of proinflammatory cytokines. Hearts from metformin‐treated mice exhibited lower expression of *Il6*, *Il1b*, and *Tnf*, although the observed differences did not reach statistical significance (Figure 2A).

**Figure 2:**
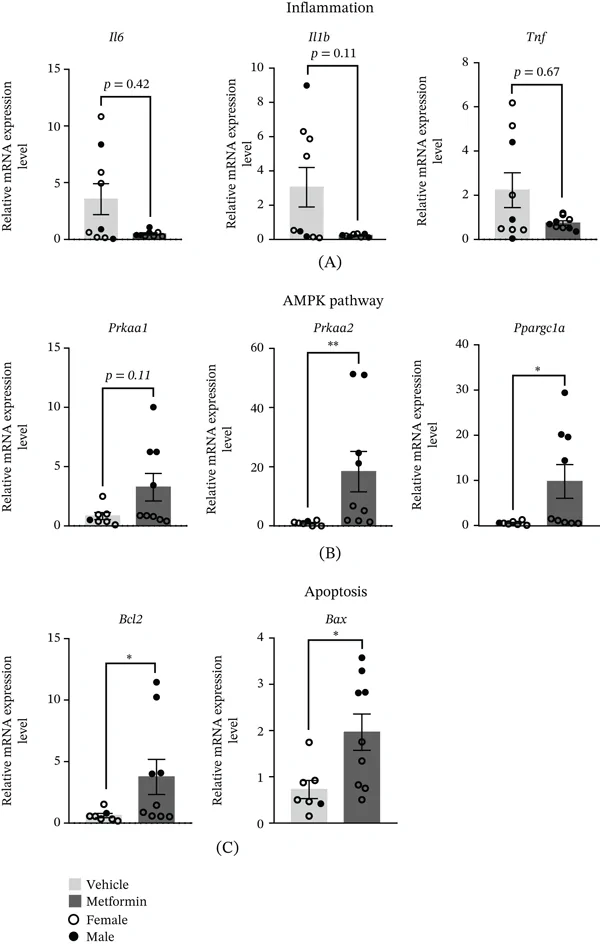
Metformin modulates AMPK signaling, apoptosis, and inflammatory pathways in autoimmune myocarditis. Relative gene expression of (A) proinflammatory cytokines, (B) AMPK pathway subunits, and (C) apoptosis proteins in the heart tissue at Day 21 of EAM. Gene expression was normalized to *Ef2*. The values are expressed as mean ± SEM, with each dot representing an individual mouse (*n* = 7–9). *p* values were calculated using the nonparametric Mann–Whitney test, except for *Bax*, which was analyzed with parametric unpaired Student′s *t*‐test.  ^∗^ *p* < 0.05,  ^∗∗^ *p* < 0.01.

To further elucidate the potential mechanism of action, we examined the expression of genes related to AMPK signaling, a known target of metformin. Transcripts for *Prkaa1*, *Prkaa2*, and *Ppargc1a* were elevated in hearts of metformin‐treated mice, consistent with activation of the AMPK metabolic stress response pathway (Figure 2B). In parallel, increased expression of proapoptotic and antiapoptotic regulators *Bax* and *Bcl2* was observed during myocardial inflammation (Figure 2C).

### 3.2. Metformin Improves Cardiac Function

In the chronic phase of EAM, the disease often progresses toward DCM, a pathological state characterized by myocardial fibrosis, ventricular remodeling, and progressive deterioration of cardiac function. To investigate whether metformin exhibits antifibrotic and cardioprotective effects in this setting, mice received the drug in drinking water according to two distinct protocols: a preventive approach (from Days 0 to 40) and a therapeutic approach (from Days 21 to 40). Control animals received regular drinking water without metformin throughout the entire experiment (Figure 3A). At Day 40, which corresponds to the DCM phase, we observed no significant differences in HW/TL or HW/BW between the treated and control groups (Figure 3B). To further assess left ventricular function and evaluate the functional impact of metformin treatment, transthoracic echocardiography was performed. Mice receiving metformin preventively exhibited a statistically significant improvement in EF, reflecting improved global systolic function, and FS, a sensitive indicator of left ventricular contractility during systole compared with vehicle‐treated mice. For EF, the overall one‐way ANOVA across the three groups indicated a trend only (*p* = 0.07; Table 1), whereas the preplanned pairwise comparison of the preventive metformin group with the vehicle group reached statistical significance in Dunnett′s post hoc test (*p* < 0.05; Figure 4B). Similar improvements were also observed in the left ventricular systolic area (LV Area s) and LV Vol s, further supporting enhanced systolic function. In contrast, therapeutic approach has no effect on improving these parameters. Other echocardiographic parameters did not show significant overall differences between the analyzed groups (Figure 4A,B; Table 1).

**Figure 3:**
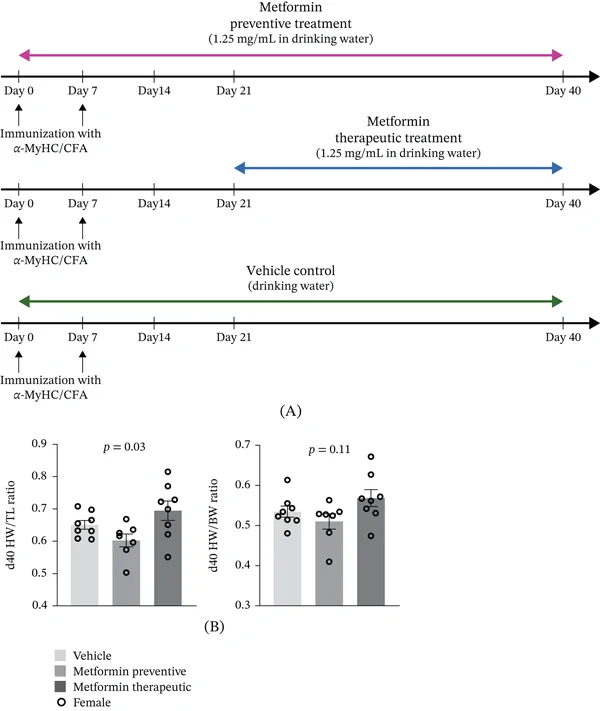
Long‐term metformin treatment does not alter body weight or heart weight in mice. (A) Schematic representation of the experimental design. (B) Heart weight (HW) to body weight (BW) ratio and heart weight (HW) to tibia length (TL) ratio. The values are expressed as mean ± SEM, with each dot representing an individual mouse (*n* = 7–8). *p* values were calculated using one‐way ANOVA followed by Dunnett′s multiple comparisons post hoc test.

**Table 1:** Echocardiographic parameters of metformin‐ and vehicle‐treated mice at Day 40 of the EAM.

Variables	d40 vehicle	d40 metformin preventive	d40 metformin therapeutic	*p*
(units, axis)	(*m* *e* *a* *n* ± *S* *E* *M*)	(*m* *e* *a* *n* ± *S* *E* *M*)	(*m* *e* *a* *n* ± *S* *E* *M*)	
Area s [mm^2^, PLAX]	12.38 ± 0.65	10.31 ± 0.21	11.07 ± 0.45	*p* = 0.02
Area d [mm^2^, PLAX]	18.36 ± 0.60	17.10 ± 0.24	17.71 ± 0.48	*p* = 0.21
LV Vol s [*μ*L, PLAX]	25.59 ± 2.51	19.04 ± 0.89	21.05 ± 1.45	*p* = 0.05
LV Vol d [*μ*L, PLAX]	47.08 ± 2.82	41.67 ± 0.89	43.09 ± 1.85	*p* = 0.19
FS [%, PLAX]	16.21 ± 1.76	19.03 ± 1.50	19.81 ± 1.55	*p* = 0.03
EF [%, PLAX]	46.14 ± 2.62	54.32 ± 1.76	51.22 ± 2.38	*p* = 0.07
SV [*μ*L, PLAX]	21.49 ± 1.40	22.63 ± 0.85	22.03 ± 1.31	*p* = 0.21
CO [mL/min, PLAX]	8.31 ± 0.90	9.17 ± 0.37	8.17 ± 0.60	*p* = 0.69
LVID s [mm, SAX]	2.49 ± 0.08	2.37 ± 0.10	2.48 ± 0.07	*p* = 0.57
LVID d [mm, SAX]	3.61 ± 0.08	3.44 ± 0.12	3.52 ± 0.08	*p* = 0.46
LVPW s [mm, SAX]	0.85 ± 0.05	0.78 ± 0.04	0.83 ± 0.05	*p* = 0.36
LVPW d [mm, SAX]	0.73 ± 0.04	0.65 ± 0.05	0.65 ± 0.03	*p* = 0.60
LVAW s [mm, SAX]	1.31 ± 0.08	1.36 ± 0.07	1.32 ± 0.08	*p* = 0.87
LVAW d [mm, SAX]	0.86 ± 0.07	0.93 ± 0.11	0.92 ± 0.08	*p* = 0.84
E/A ratio [MV flow, A4C]	2.07 ± 0.21	2.10 ± 0.39	1.74 ± 0.10	*p* = 0.47

*Note:* The values are expressed as mean ± SEM (*n* = 7–8). The *p* values shown in the table represent overall differences among experimental groups and were calculated using parametric one‐way ANOVA or, for CO, E/A ratio, and LVPW s, the nonparametric Kruskal–Wallis test. Pairwise post hoc comparisons for EF, FS, Area s and LV volume s for selected groups are presented in Figure 4. These overall *p* values do not incorporate the post hoc comparisons: for EF the overall ANOVA *p* value was 0.07, whereas the preplanned comparison between the vehicle‐ and preventive metformin‐treated groups was significant in Dunnett′s test (*p* < 0.05; Figure 4).

Abbreviations: A, peak Doppler blood inflow velocity across the mitral valve during late diastole; Area d, left ventricular surface in the diastolic phase; Area s, left ventricular surface in the systolic phase; CO, cardiac output; E, peak Doppler blood inflow velocity across the mitral valve during early diastole; EF, ejection fraction; FS, fractional shortening; LVAW d, left ventricular end‐diastolic anterior wall thickness; LVAW s, left ventricular end‐systolic anterior wall thickness; LVID d, left ventricular end‐diastolic diameter; LVID s, left ventricular end‐systolic diameter; LVPW d, left ventricular end‐diastolic posterior wall thickness; LVPW s, left ventricular end‐systolic posterior wall thickness; LV Vol d, left ventricular end‐diastolic volume; LV Vol s, left ventricular end‐systolic volume; PLAX, parasternal long axis; SAX, parasternal short axis; SV, stroke volume.

**Figure 4:**
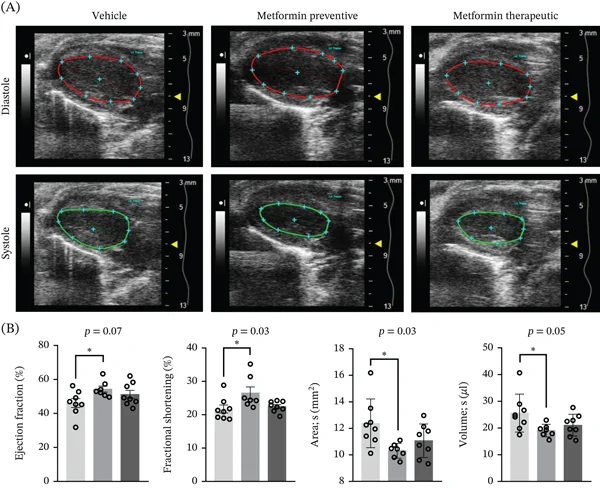
Preventive metformin administration preserves cardiac function at Day 40. (A) Representative echocardiographic PLAX heart projections of metformin‐ and vehicle‐treated mice at diastole and systole at Day 40 of the EAM. (B) Selected echocardiographic parameters. The full list of echocardiographic parameters is presented in Table 1. The values are expressed as mean ± SEM, with each dot representing an individual mouse (*n* = 7–8). Overall *p* values for differences among experimental groups were calculated using one‐way ANOVA and are indicated above the respective plots. Pairwise comparisons with the vehicle‐treated group were performed using Dunnett′s multiple‐comparisons test, with statistical significance indicated above compared groups,  ^∗^ *p* < 0.05. Dunnett′s test controls the family‐wise error rate for these preplanned comparisons and was therefore applied independently of the outcome of the overall ANOVA; accordingly, for EF a significant pairwise difference between the vehicle‐ and preventive metformin‐treated groups was detected despite a nonsignificant overall ANOVA *p* value (*p* = 0.07; Table 1).

### 3.3. Metformin Limits Cardiac Hypertrophy but Does Not Affect Cardiac Fibrosis

Since myocardial remodeling and fibrosis are a hallmark of the DCM phenotype, we analyzed changes on tissue and molecular level. Masson′s trichrome and Sirius red staining revealed substantial ECM deposition indicative of fibrosis in all groups at Day 40, with no significant reduction in collagen deposition in metformin‐treated mice compared with controls. Similarly, immunohistochemical staining for vimentin, a cytoskeletal marker of mesenchymal stromal cells, showed no measurable impact of metformin (Figure 5A). At the molecular level, qPCR analysis of profibrotic markers mostly confirmed the histological findings. Expression of key fibrosis‐associated genes, including *Col3a1*, *Postn*, and *Acta2*, was not affected in metformin‐treated mice at Day 40 of EAM. Remarkably, *Col1a1* expression was increased in the preventive metformin group compared with controls, whereas in the therapeutic group, expression levels remained similar to those of control mice (Figure 5B).

**Figure 5:**
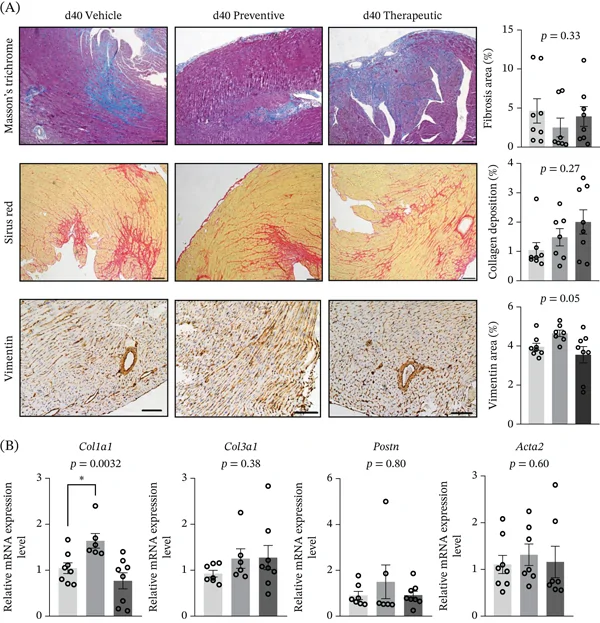
Metformin treatment does not significantly reduce myocardial fibrosis in autoimmune myocarditis. (A) Representative histology showing Masson′s trichrome and Sirius red staining, as well as immunohistochemical staining for vimentin along with quantification at Day 40 of EAM. Scale bars = 100 *μ*m. (B) Relative gene expression of profibrotic markers in the heart tissue at Day 40 of EAM. Genes expression was normalized to *Ef2*. The values are expressed as mean ± SEM, with each dot representing an individual mouse (*n* = 6–8). Overall *p* values for differences among experimental groups were calculated using nonparametric Kruskal–Wallis, except for vimentin area, which was analyzed with parametric one‐way ANOVA, and are indicated above the respective plots. Pairwise comparisons with the vehicle‐treated group were performed using Dunn′s (nonparametric) or Dunnett′s (parametric) multiple‐comparisons test, with statistical significance indicated above compared groups,  ^∗^ *p* < 0.05.

Cardiomyocyte remodeling is another critical feature of DCM, characterized by alterations in cell size, structure, and function. This process often involves hypertrophy, cytoskeletal disorganization, impaired contractility, and changes in gene expression profiles that collectively contribute to cardiac dysfunction. To assess these changes, we evaluated morphological and molecular markers of cardiomyocyte remodeling in both control and metformin‐treated groups. WGA staining was used to assess cardiomyocyte cross‐sectional area at Day 40. Preventive metformin treatment significantly attenuated cardiomyocyte enlargement compared with the control group (Figure 6A). At the gene expression level, cardiomyocyte hypertrophy is often accompanied by reactivation of the fetal gene program, as for example upregulation of *Acta1* expression. In metformin‐treated mice expression of *Acta1* was reduced. Moreover, preventive treatment with metformin was associated with elevated expression level of *Myh6*, a marker characteristic of mature cardiomyocytes. In contrast, no significant changes were detected in the expression of *Myh7b* or *Nppb* (Figure 6B).

**Figure 6:**
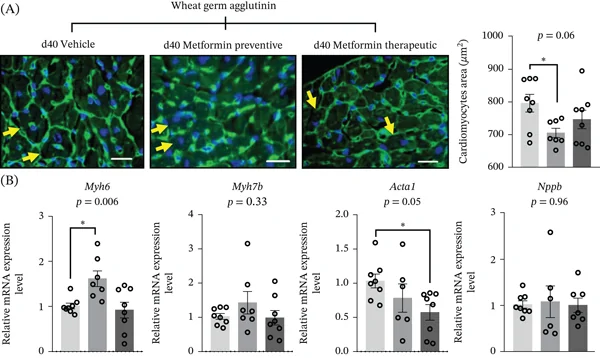
Metformin prevents cardiac hypertrophy, reducing cardiomyocyte cross‐sectional area and hypertrophy‐associated gene expression. (A) Representative immunofluorescence showing wheat germ agglutinin staining along with quantification of cardiomyocyte cross‐sectional area. Scale bars = 10 *μ*m. (B) Relative gene expression of cardiomyocyte hypertrophic markers in the heart tissue at Day 40 of EAM. Gene expression was normalized to *Ef2*. The values are expressed as mean ± SEM, with each dot representing an individual mouse (*n* = 6–8). Overall *p* values for differences among experimental groups were calculated using parametric one‐way ANOVA, except for *Acta1,* which was analyzed with nonparametric Kruskal–Wallis, and are indicated above the respective plots. Pairwise comparisons with the vehicle‐treated group were performed using Dunn′s (nonparametric) or Dunnett′s (parametric) multiple‐comparisons test, with statistical significance indicated above compared groups,  ^∗^ *p* < 0.05.

Collectively, these results demonstrate that metformin, when administered preventively, may attenuate selected aspects of cardiac remodeling occurring in the transition from autoimmune myocarditis to DCM. Early metformin treatment preserves systolic function and suppresses cardiomyocyte hypertrophy, underscoring its potential as a cardioprotective agent in inflammatory heart disease.

## 4. Discussion

Metformin is a widely prescribed drug, primarily recognized for its ability to reduce insulin resistance; however, its therapeutic profile is continuously expanding. Experimental and clinical studies indicate that metformin exerts beneficial effects well beyond glycemic control, with potential applications in oncology [9, 10], neurodegenerative disorders [3, 4], polycystic ovary syndrome [7, 8], and cardiovascular disease [16, 47]. At present, little is known about the effects of metformin in inflammatory heart disease. Although previous investigations have largely focused on its benefits in systemic infectious models, including sepsis‐ and endotoxin‐induced myocarditis [21, 48], our findings provide the first evidence of its effects in a heart‐specific autoimmune myocarditis model. Moreover, they highlight a context of cardiac‐restricted inflammation and postinflammatory DCM that remains comparatively underexplored.

Myocarditis is characterized by extensive inflammatory infiltration of the myocardium, predominantly by myeloid and T cells, accompanied by elevated production of proinflammatory cytokines such as TNF‐*α*, IL‐1*β*, and IL‐6, which contribute to myocardial injury [49]. In our model, vehicle‐treated Balb/c mice at Day 21 exhibited the expected upregulation of inflammatory markers, consistent with established disease mechanisms. Metformin administration exerted a protective effect, although this was less pronounced than reported in earlier studies. For example, in sepsis‐induced myocarditis in zebrafish [48] and LPS‐induced myocarditis in mice [21], metformin markedly reduced leukocyte infiltration and cytokine production. In contrast, in our EAM model we observed only a nonsignificant trend toward such improvements, suggesting a more moderate therapeutic response. This divergence likely reflects differences in the nature of inflammation; prior studies predominantly represent systemic inflammatory conditions, whereas our findings derive from a heart‐specific autoimmune model, indicating that the observed cardioprotective effects may be mediated by mechanisms unique to organ‐restricted inflammation. Furthermore, the zebrafish model is substantially different from mice in terms of immune complexity, pharmacokinetics, and disease progression, further underscoring the context‐dependency of metformin′s effects.

The molecular mechanisms underlying metformin′s cardioprotective action remain the subject of active investigation. Current evidence suggests that activation of the AMPK pathway may contribute to the observed protective effects. Stimulation of this cascade has been shown to suppress the expression of proinflammatory mediators [17, 21, 50], reduce oxidative stress [51, 52], and improve cardiac performance [53, 54]. Consistent with these reports, our data demonstrate that metformin significantly increased mRNA expression of *Prkaa2* and *Ppargc1a*, encoding AMPK and PGC‐1*α*, two key regulators of energy metabolism that functionally interact. Although our findings support the involvement of AMPK‐dependent signaling pathways, direct validation of its functional contribution was not demonstrated in this study. However, studies in other heart failure models using the selective AMPK inhibitor Compound C, AMPK*α* knockout mice, and AMPK kinase‐dead models demonstrated a loss of anti‐inflammatory and antifibrotic effects, supporting cardioprotective role of AMPK signaling [55–58].

Furthermore, we observed upregulation of both *Bax* and *Bcl2* in metformin‐treated mice, suggesting that the drug also modulates apoptotic pathways during myocardial inflammation. Similar findings have been reported in rat cardiac myoblasts, where LPS stimulation induced *Bax* expression and suppressed *Bcl2*, lowering the Bcl2/Bax ratio and promoting apoptosis. This process was mitigated by metformin, which restored the ratio in favor of cell survival [59]. The concurrent increase in both proapoptotic and antiapoptotic markers in our study may reflect a compensatory response, wherein cells activate survival pathways to counterbalance proapoptotic signaling and preserve viability.

In the chronic phase of EAM, the acute inflammatory response subsides, giving rise to postinflammatory DCM characterized by extensive myocardial fibrosis and cardiac dysfunction [43, 60]. In our model, mice that received metformin in their drinking water from Day 0 until Day 40 exhibited improved left ventricular function compared with vehicle‐treated mice or those given metformin only from the onset of inflammation on Day 21. These functional benefits were supported by the analysis of cardiomyocyte cross‐sectional area, which demonstrated reduced hypertrophy in the preventive group. At the molecular level, metformin treatment modulated reactivation of the fetal gene program in cardiomyocytes, a hallmark of maladaptive cardiac remodeling, reflected by decreased expression of *Acta1* and increased expression of *Myh6*, thereby confirming its antihypertrophic effect.

Fibrosis represents a common pathological endpoint across multiple cardiac diseases, irrespective of the initiating factor. Accumulating evidence indicates that metformin exerts robust antifibrotic effects in diverse experimental models of cardiac injury. In metabolic settings, beneficial outcomes have been demonstrated in hyperhomocysteinemia‐induced cardiac hypertrophy [19] and various forms of obesity‐related cardiac injury [20, 61, 62]. Comparable protective effects have been reported in structural and ischemic injury models, including postinfarction myocardial fibrosis [18] and DCM of nonischemic origin [63, 64]. In contrast, such antifibrotic activity was not evident under the applied experimental conditions in the EAM model, as we did not detect significant changes in collagen deposition, the major extracellular matrix component, either histologically or at the molecular level. Importantly, this finding should not be interpreted as evidence that metformin lacks antifibrotic activity. ECM remodeling is a complex and dynamic process, and our results describe the phenotype at a single time point only. The absence of an evident antifibrotic effect observed in the present study may be related to insufficient dosing or to the relatively early stage of fibrosis in our model.

A limitation of this study is that metformin was administered via drinking water, which does not allow precise control of the dose received by each individual animal. Although individual water intake was not measured, comparable body weight changes in the metformin‐treated group suggest similar water consumption and, consequently, relatively consistent drug exposure across animals. In addition, this study was limited to investigating the effects of metformin in the EAM model and did not include a healthy control group. Furthermore, given the inherent biological variability of the EAM model, we restricted the study population to female mice and assessed the effects at Day 40 to limit group sizes. Consequently, the generalizability of our findings is currently limited to female mice at this time point.

Collectively, our findings indicate that metformin exerts greater efficacy during the early stages of disease progression than after the establishment of postinflammatory cardiac remodeling. These observations may suggest that metformin‐mediated cardioprotection is driven predominantly by suppression of early inflammatory signaling and hypertrophic pathways, whereas reversal of established structural remodeling and fibrosis may require higher dosing or more intensive therapeutic approaches.

Taken together, our data demonstrate for the first time the cardioprotective role of metformin in autoimmune myocarditis and postinflammatory DCM in the EAM mouse model. The findings highlight the importance of initiating treatment at an early stage of disease progression, before irreversible structural remodeling has occurred. In this context, metformin’s beneficial effects appear to extend well beyond its established metabolic actions, encompassing the modulation of inflammatory signaling, regulation of energy metabolism, and apoptotic pathways. Such pleiotropic activity suggests that metformin may act at multiple levels of the pathological cascade, potentially influencing both immune‐mediated injury and the downstream remodeling processes that lead to DCM. Nevertheless, the lack of detectable changes in myocardial collagen content underscores the complexity of myocardial repair, in which restoration of contractile performance may precede, and possibly occur independently of, ECM remodeling. These findings underscore the need for future studies to clarify the cause‐and‐effect relationship between inflammatory resolution, functional recovery, and extracellular matrix remodeling, as well as to determine the optimal timing, duration, and dosing strategies required to maximize the therapeutic efficacy of metformin in postinflammatory cardiac disease.

**Nomenclature**
*α*MyHC: *α*‐myosin heavy chain
A: peak Doppler blood inflow velocity across the mitral valve during late diastole
A4C: apical four‐chamber views
AMPK: AMP‐activated protein kinase
Area d/s: left ventricular surface in the diastolic/systolic phase
BPM: beats per minute
CFA: complete Freund’s adjuvant
CO: cardiac output
DAB: diaminobenzidine
DCM: dilated cardiomyopathy
E: peak Doppler blood inflow velocity across the mitral valve during early diastole
EAM: experimental autoimmune myocarditis
EF: ejection fraction
FS: fractional shortening
H&E: hematoxylin and eosin
HR: heart rate
HW/BW: heart weight to body weight ratio
HW/TL: heart weight to tibia length ratio
LVAW d/s: left ventricular end‐diastolic/end‐systolic anterior wall thickness
LVID d/s: left ventricular end‐diastolic/end‐systolic diameter
LVPW d/s: left ventricular end‐diastolic/end‐systolic posterior wall thickness
LV Vol d/s: left ventricular end‐diastolic/end‐systolic volume
PBS: phosphate‐buffered saline
PLAX: parasternal long‐axis
qPCR: quantitative polymerase chain reaction
SAX: parasternal short‐axis
SEM: standard error of the mean
SV: stroke volume
WGA: wheat germ agglutinin

## Author Contributions

**Magdalena Czepiec:** methodology, formal analysis, investigation, writing – original draft, validation, visualization, data curation, revising. **Katarzyna Fryt:** methodology, investigation. **Agnieszka Jaźwa-Kusior:** methodology. **Andrea Laimbacher:** methodology. **Gabriela Kania:** resources. **Maciej Siedlar:** resources. **Małgorzata Tyszka-Czochara:** conceptualization, supervision. **Przemysław Błyszczuk:** conceptualization, supervision, writing – review and editing, funding acquisition, revising.

## Funding

The study was supported by the National Science Centre, Poland (10.13039/501100004281, Grant Number 2019/35/B/NZ5/00551).

## Conflicts of Interest

The authors declare no conflicts of interest.

## Data Availability

The data that support the findings of this study are available from the corresponding author upon reasonable request.
